# Supplementary material for: The thresholding problem and variability in the EEG graph network parameters
Source: Sci Rep. 2022 Nov 4;12:18659. doi: 10.1038/s41598-022-22079-2 (PMC9636266; doi:10.1038/s41598-022-22079-2)
Supplement: Supplementary file 1 — Supplementary Information. [file 41598_2022_22079_MOESM1_ESM.docx]

**Appendix**


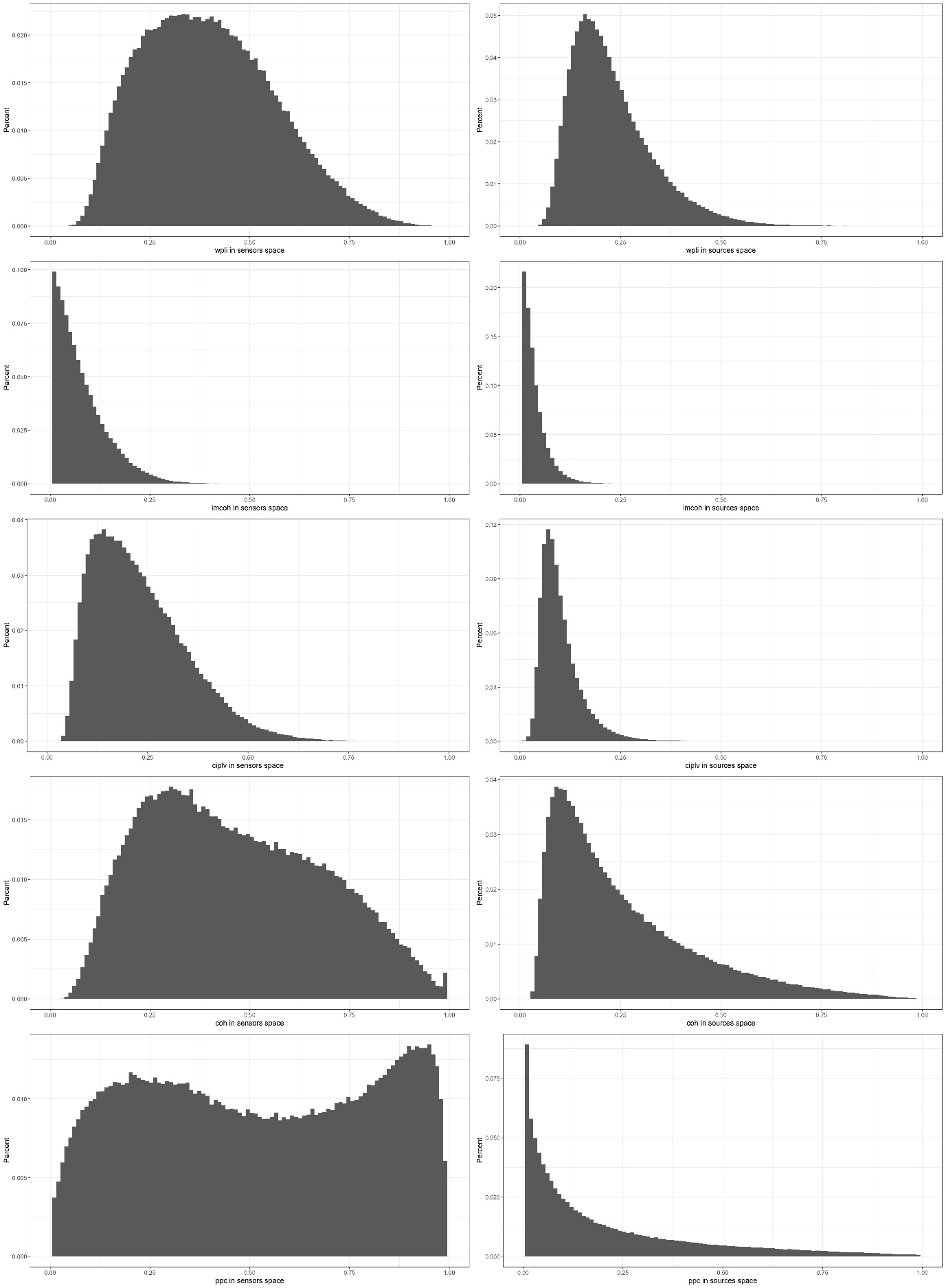


Supplementary figure 1. Distributions of synchronization measures


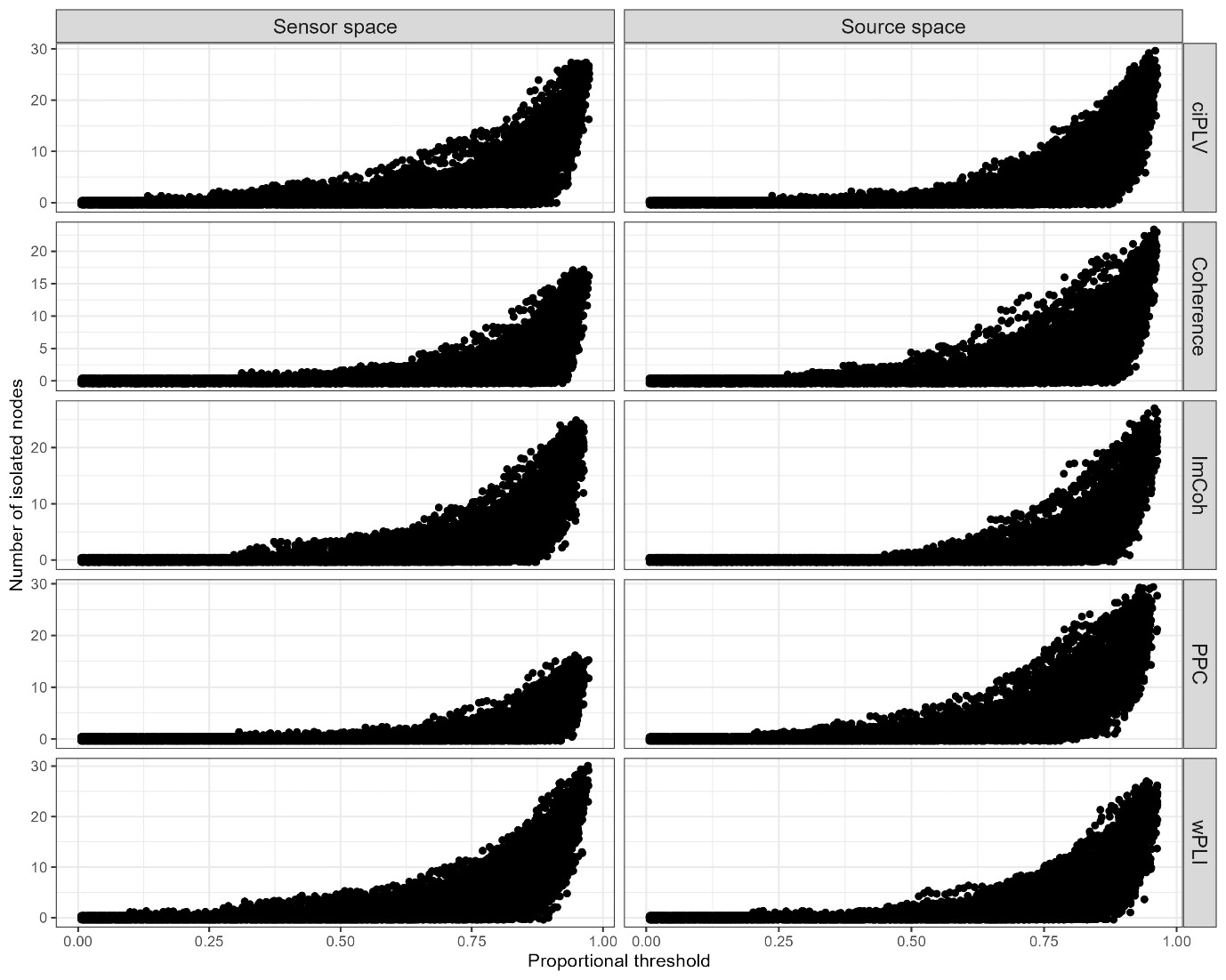


Supplementary figure 2. A number of isolated nodes across different thresholds. Each node represents one matrix.


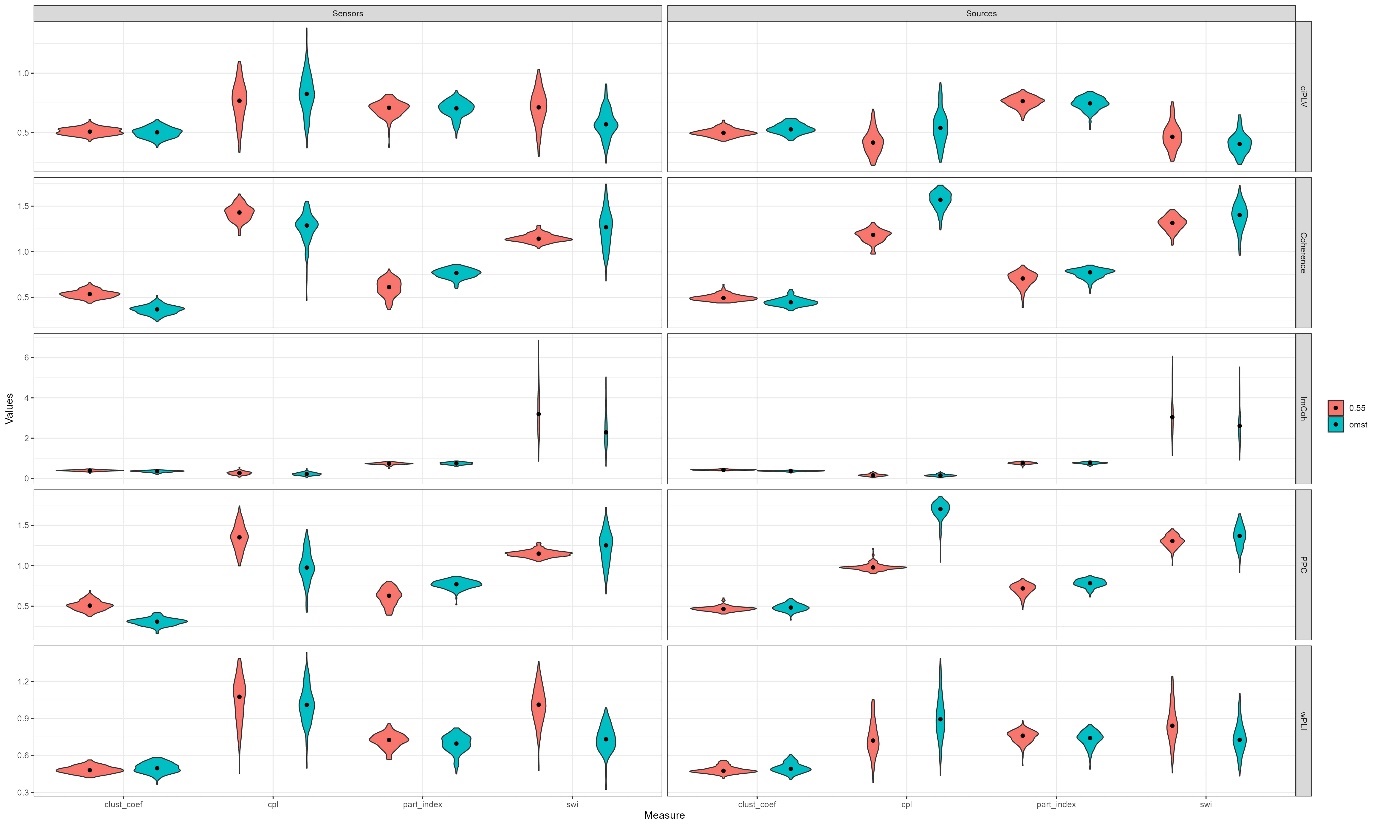


Supplementary figure 3. Comparison of the measures derived from the thresholded graph (red) and OMST-graph (blue).
